# Clinical characteristics of hereditary spherocytosis with red blood cell membrane protein gene variants

**DOI:** 10.3389/fped.2025.1523288

**Published:** 2025-02-10

**Authors:** Jingying Cheng, Liqiang Zhang, Jiafeng Yao, Shasha Zhao, Jin Jiang

**Affiliations:** Department of Hematology, Beijing Children’s Hospital, Capital Medical University, National Center for Children’s Health, Beijing, China

**Keywords:** hereditary spherocytosis, red blood cell membrane protein, pathogenic variants, splenectomy, genetic testing

## Abstract

The clinical manifestations of hereditary spherocytosis (HS) are often heterogeneous, spanning from asymptomatic to severe symptoms that may pose life-threatening risks. Genotype-phenotype correlations remain controversial in clinical research. This retrospective study evaluated the correlation between genetic variants and clinical characteristics in a cohort of 64 Chinese pediatric patients with HS. The predominant variants were found in the *ANK1* (27 cases, 42%) and *SPTB* (26 cases, 41%) genes, while variants in the *SPTA1* (6 cases, 9%) and *SLAC4A1* genes (5 cases, 8%) were less common. No *EPB42* variants were detected. A total of 71 variants were identified. Variation types included nonsense (21%), missense (27%), frameshift mutations (39%), splicing (8%), and large fragment deletions (4%). No statistical differences in hemoglobin levels, MCV, MCH, MCHC, or reticulocytes were observed across the various genetic variant groups. Bilirubin levels were remarkably elevated in patients with HS variants, and those with *SPTB*-HS had significantly higher bilirubin levels, including total bilirubin (*p* = 0.033) and indirect bilirubin (*p* = 0.018) compared to those with *SPTA1*-HS. Moreover, those with the *ANK1* variants displayed reduced resistance to lysis at varying NaCl concentrations in comparison to those with the *SPTA1* variants (*p* = 0.047). In short, patients with the *ANK1* and *SPTB* variants had the most severe disease, while those with the *SPTA1* variants had the mildest. Genetic testing is advised in patients without a family history or who are difficult to diagnose with routine laboratory tests, as this may also provide references for clinical treatment and genetic counseling.

## Introduction

Hereditary spherocytosis (HS) is a prevalent genetic disorder characterized primarily by hemolytic anemia, jaundice, splenomegaly, spherocytosis, and a family history of hemolytic anemia. Because of abnormalities in the coding of red blood cell membrane skeleton protein genes, the corresponding membrane skeleton protein synthesis is reduced or defective, leading to partial loss of the red blood cell membrane and the red blood cells becoming spherical. They are then sequestered and phagocytosed by the spleen for clearance (1). Pathogenic variation mainly included the *ANK1*, *SPTA1*, *SPTB*, *SLC4A1*, and *EPB42* genes, encoding ankyrin, α-spectrin, β-spectrin, band 3 protein, and 4.2 protein, respectively (2, 3). Diagnosis of HS typically relies on clinical manifestations, family history, and a series of laboratory tests, which may present false-negative and false-positive results. The clinical manifestations of HS vary greatly, spanning from asymptomatic to severe symptoms that may pose life-threatening risks. Some children with mild disease may not be diagnosed until the occurrence of infections, and others are diagnosed serendipitously during treatment for other diseases. Therefore, the diagnosis of HS poses a challenge, especially for asymptomatic or atypical cases that rely solely on clinical presentation, family history, and hematologic laboratory tests.

With the widespread application of genetic diagnostic technologies, genetic testing offers enhanced diagnostic efficiency and rapidly provides a comprehensive and detailed genetic analysis in patients suspected of HS. Furthermore, early genetic testing can aid in the understanding of phenotypes and inheritance patterns, which also helps in risk assessment and genetic counseling. Initial studies suggested that genetic heterogeneity forms the basis of clinical heterogeneity (4). However, studies on HS in recent decades have been controversial, with most studies suggesting that pediatric patients with *SPTA1* variants exhibit more severe anemia, while patients with *SLC4A1* mutations present milder phenotypes, such as higher hemoglobin levels and lower reticulocyte counts (5, 6). Researchers from the Netherlands argue that variants in *ANK1* and *SPTB* may lead to heightened severity of HS manifestations in contrast to *SPTA1*, with these patients typically having lower hemoglobin and higher reticulocyte levels. However, no significant difference between *ANK1* and *SPTB* was observed (7). Research in China on this topic remains scarce, especially in children (8, 9). HS prevalence in China is approximately 1.27 per 100,000 in males and 1.49 per 100,000 in females, most of whom are diagnosed during childhood (10). Due to the significant heterogeneity in clinical manifestations in children with HS, it may be difficult to diagnose those with mild symptoms and those who are frequently transfused with routine laboratory examinations. In this study, we retrospectively summarized and analyzed the clinical and genetic characteristics of 64 Chinese children with HS, investigating the impact of different gene variant types on clinical presentation.

## Methods

### Study participants

The clinical characteristics of 64 pediatric patients with HS admitted to Beijing Children's Hospital, Capital Medical University from January 2018 to December 2023 were collected for retrospective analysis. Patients were included if they had a clinical diagnosis of HS (based on medical history, non-immune hemolytic anemia, splenomegaly, and spherocytes on morphology) (2) and had genetic testing (HS variation was identified) performed. Informed consent was obtained from the patients' parents. The study was approved by the Ethics Committee of Beijing Children's Hospital.

### Data collection

We retrospectively collected data on patient demographics, family history, data of splenomegaly by abdominal ultrasound, jaundice, anemia, or cholecystolithiasis, laboratory testing data [including blood smears reviewed by hematologist, complete blood count, blood biochemical indexes, and the oxidative fermentative (OF) test (11)], treatments, and therapeutic effects.

### Genetic testing

All genetic diagnoses in this study were obtained using targeted-next-generation sequencing (NGS) (a genetic panel of the blood system) or whole-exome sequencing (WES) and were validated using Sanger sequencing. *REVEL* software was used to predict protein structure-function for unreported novel variants, and pathogenicity analysis was conducted according to the Variant Interpretation Guidelines released by the American College of Medical Genetics and Genomics (ACMG), excluding single nucleotide polymorphisms.

### Statistical analysis

Statistical analysis was conducted using SPSS software (version 23.0). Categorical data are expressed as frequencies and percentages, and normally distributed continuous data are presented as mean ± standard deviation. Paired *t*-tests were used to compare normally distributed data, while the Kruskal–Wallis test was used for non-normally distributed continuous data. Pairwise comparisons between groups were performed using the Mann–Whitney *U* test, with *p* < 0.05 considered statistically significant.

## Results

### General information

This study comprised 64 participants, consisting of 34 males and 30 females, with a median age of 3.5 years (range: 0.1–14.6 years). The age of onset ranged from immediately after birth to 12 years, with 46 (72%) having disease onset before 1 year of age. The number of patients who had the disease decreased with increasing age [≥3 years and <7 years, 9 (14%); ≥7 years and <12 years, 3 (5%); ≥12 years, 2 (3%);]. Moreover, 21 patients (33%) had a family history of HS, and 4 of them had parents who underwent a splenectomy. Fifty-five (86%) had a history of neonatal jaundice. Overall, the symptoms were mild, and only four patients underwent exchange transfusion therapy. All patients had undergone abdominal ultrasound. Splenomegaly was present in 49 cases (77%), hepatomegaly in 37 (57%), and cholelithiasis in 9 (14%). Among the 64 patients, approximately two-thirds had received red blood cell transfusions, and 14 (22%, 6 cases of *ANK1,* and 6 cases of *SPTB*) developed red blood cell transfusion dependence. In this study, all 64 children had a clinical diagnosis of HS and underwent genetic testing, including 57 using targeted NGS and 7 using WES. The detailed clinical features of these patients with the different genotypes are presented in Table 1.

**Table 1:** Baseline and clinical characteristics of 64 HS children with different gene variants.

	SPTB (*n* = 26)	ANK1 (*n* = 27)	SLC4A1 (*n* = 5)	SPTA1 (*n* = 6)	*P* value
Baseline characteristics					
Gender, male (%)	11 (42%)	17 (63%)	4 (80%)	2 (33%)	—
Age (years), media (range)	4.3 (0.1–12.9)	3.3 (0.1–14.6)	4.4 (0.1–12.1)	0.7 (0.2–6.6)	0.793
Family history (*n*, %)	10 (38%)	6 (22%)	3 (60%)	2 (33%)	
Clinical characteristics					
Neonatal jaundice (*n*, %)	23 (88%)	24 (89%)	5 (100%)	3 (50%)	
Neonatal transfusion/exchange transfusion (*n*, %)	10 (38%)	7 (26%)	0 (0%)	1 (17%)	
Splenomegaly (*n*, %)	19 (73%)	23 (85%)	3 (60%)	4 (67%)	
Cholelithiasis (*n*, %)	3 (12%)	5 (19%)	1 (20%)	0 (0%)	
Transfusion (≥1 red cell transfusion) (*n*, %)	14 (54%)	19 (70%)	3 (60%)	4 (67%)	
Transfusion dependence (*n*, %)	6 (23%)	6 (22%)	1 (20%)	1 (17%)	

### Gene variants

Variant data of the 64 patients with HS are shown in Table 2. All patients had heterozygous or compound heterozygous variants. *ANK1* and *SPTB* variants were predominant in all patients, with 27 cases (42%) of *ANK1* variants, 26 (41%) of *SPTB* variants, six (9%) of *SPTA1* variants, and five (8%) of *SLAC4A1* variants. No *EPB42* variants were detected. The sources of variation showed 24 cases (38%) of inherited variants from parents, 35 (55%) were spontaneous, and 5 patients’ fathers or mothers were not sampled. Almost all patients exhibited an autosomal dominant inheritance pattern. In our study, 71 variants were identified, among which 59 variants have not been previously reported. Variation types included 15 cases (21%) of nonsense, 19 (27%) of missense, 28 (39%) of frameshift mutations, 6 (8%) of splicing, and 3 (4%) of large fragment deletions. The *ANK1* c.127-3C>G (splicing) variant was found in both patients 17 and 61. Five patients had more than one variant. Secondary *SPTA1* variants were the most common (3 cases), but these 5 secondary variants were unlikely to be disease-causing according to ACMG guidelines. Regretfully, for *SPTA1,* the α*^LELY^* allele and α*^LEPRA^* allele were not investigated.

**Table 2:** Complete list of causative variants in the cohort.

No.	Gene	Allele	Protein effect	Variant classification	Sources of variation	Variation type	Inheritance pattern	2nd variant	Variant classification
1	ANK1	c.2926C>T	p. R976X	P	Spontaneous mutation	Nonsense mutation	AD		
2	SPTB	c.3824_3825del	p. E1275fs	LP	Father	Frameshift mutation	AD		
3	SPTB	c.1A>G	p. M1V	P	Spontaneous mutation	Missense mutation	AD	EPB4	VUS
4	ANK1	c.3451_3459AGCT	p. A1151Sfs*	P	Unknown	Frameshift mutation	AD		
5	SPTA1	c.928G>A	p. E310K	VUS	Father	Missense mutation	AD/AR		
		c.676G>A	p. E226K	VUS	Mother	Missense mutation	AD/AR		
6	ANK1	c.367C>T	p. Q123X	P	Spontaneous mutation	Nonsense mutation	AD		
7	ANK1	c.3604delG	p. D1202Tfs*28	P	Spontaneous mutation	Frameshift mutation	AD		
8	SPTB	c.2245C>T	p. Q749X	P	Father	Nonsense mutation	AD		
9	SPTB	c.5221delG	p. A1741Rfs*10	P	Mother	Frameshift mutation	AD		
10	SLC4A1	c.2279G>A	p. R760Q	LP	Mother	Missense mutation	AD/AR	SPTA1	VUS
11	SPTA1	c.2659C>T	p. R887X	LP	Mother	Nonsense mutation	AD/AR		
12	SPTA1	c.82C>A	p. R28S	P	Spontaneous mutation	Missense mutation	AD/AR		
13	SPTB	c.4735C>T	p. R1579X	P	Spontaneous mutation	Nonsense mutation	AD		
14	SLC4A1	c.2386G>A	p. G796R	LP	Mother	Missense mutation	AD/AR		
15	ANK1	c.4429C>T	p. R1477X	P	Spontaneous mutation	Nonsense mutation	AD		
16	ANK1	c.3100_3122del	p. I1034Pfs*116	LP	Father	Frameshift mutation	AD	SPTA1	VUS
17	ANK1	c.127-3C>G	Splicing	LP	Spontaneous mutation	Splicing mutation	AD		
18	SPTB	c.555delG	p. M185Ifs*27	P	Spontaneous mutation	Frameshift mutation	AD		
19	ANK1	c.226C>T	p. Q76X	LP	Father	Nonsense mutation	AD		
20	SPTB	c.853delG	p. V285Wfs*19	LP	Mother	Frameshift mutation	AD		
21	ANK1	c.364G>A	p. G122R	LP	Spontaneous mutation	Missense mutation	AD		
22	ANK1	c.636_637insACGGCACCAAGGGGAAGGTGCGCCTCCCGGCCCTGCACATC	p. A213Tfs*87	P	Spontaneous mutation	Frameshift mutation	AD		
23	SPTB	c.4873C>T	p. R1625X	p	Spontaneous mutation	Nonsense mutation	AD		
24	SPTB	c.5773C>T	p. Q1925X	LP	Mother	Nonsense mutation	AD		
25	SPTB	c.5317_5321GACGG>CTGAACGAGATGTGG	p. 1773fs	P	Spontaneous mutation	Frameshift mutation	AD		
26	SPTB	c.560C>T	p. T187M	VUS	Mother	Missense mutation	AD		
27	ANK1	c.3327+1G>A	Splicing	LP	Unknown	Splicing mutation	AD		
		c.3713C>T	p. A1238V	VUS	Unknown	Missense mutation	AD		
28	SPTB	c.1261G>T	p. E421X	LP	Mother	Nonsense mutation	AD	SLC4A1	VUS
29	SPTB	c.5157dupG	p. Q1720Afs*4	P	Spontaneous mutation	Frameshift mutation	AD		
30	ANK1	c.2032G>T	p. E678X	P	Spontaneous mutation	Nonsense mutation	AD		
31	ANK1	c.1774delG	p. D592Tfs*12	LP	Unknown	Frameshift mutation	AD		
		c.1771C>T	p. R591W	VUS	Unknown	Missense mutation	AD		
32	ANK1	c.5138_5139delTT	p. L1713Rfs*68	P	Spontaneous mutation	Frameshift mutation	AD		
33	ANK1	c.436delA	p. T146Hfs*27	LP	Mother	Frameshift mutation	AD		
34	ANK1	c.2489_2492delTAGT	p. L830Sfs*7	P	Spontaneous mutation	Frameshift mutation	AD		
		c.394G>T	p. D132Y	VUS	Father	Missense mutation	AD		
35	SPTB	c.2805-2A>T	Splicing	P	Spontaneous mutation	Splicing mutation	AD		
36	ANK1	c.3611_3649delGGGAGGGAGACACCACCAGCCTGCGCCTGCTTTGCAGCG	p. 1204_1217del	LP	Spontaneous mutation	Deletion mutation	AD		
37	SPTA1	c.6544G>C	p. D2182H	VUS	Father	Missense mutation	AD/AR		
38	SPTB	c.3571_3572del	p. L1191Gfs*20	P	Spontaneous mutation	Frameshift mutation	AD		
39	ANK1	c.1433_1436del	p. A478Efs*41	P	Spontaneous mutation	Frameshift mutation	AD		
40	SLC4A1	c.1394C>T	p. S465l	VUS	Father	Missense mutation	AD/AR		
41	SPTA1	c.7250T>C	p. F2417S	VUS	Father	Missense mutation	AD/AR		
		c.2320C>T	p. R774X	LP	Mother	Nonsense mutation	AD/AR		
42	SPTB	c.3560A>G	p. Q1187R	LP	Spontaneous mutation	Missense mutation	AD		
43	ANK1	c.1776delC	p. D592Efs*12	LP	Unknown	Frameshift mutation	AD		
44	ANK1	c.442dupC	p. L148Pfs*14	P	Spontaneous mutation	Frameshift mutation	AD		
45	SLC4A1	c.1700delT	p. L567Rfs*20	P	Spontaneous mutation	Frameshift mutation	AD/AR		
46	SPTB	c.3320delA	p. K1107Rfs*21	LP	Unknown	Frameshift mutation	AD		
47	SPTB	c.4581delT	p. L1528Wfs*53	P	Spontaneous mutation	Frameshift mutation	AD		
48	SPTB	c.965delT	p. I322Tfs*4	P	Spontaneous mutation	Frameshift mutation	AD		
49	ANK1	c.1816dupC	p. L606Pfs* 48	P	Spontaneous mutation	Frameshift mutation	AD		
50	SPTB	c.1931G>A	p. W644X	LP	Father	Nonsense mutation	AD		
51	ANK1	c.4274delT	p. L1425Rfs*22	P	Spontaneous mutation	Frameshift mutation	AD		
52	ANK1	c.3877C>T	p. R1293X	P	Spontaneous mutation	Nonsense mutation	AD		
53	SLC4A1	c.2572_2601delGCCCTGCCCTTCGTCCTCATCCTCACTGTG	p. 858_867del	VUS	Spontaneous mutation	Deletion mutation	AD/AR		
54	SPTB	c.4405delA	p. R1469Gfs*18	P	Spontaneous mutation	Frameshift mutation	AD		
55	ANK1	c.5342_5345delAAGG	p. E1781Gfs*2	LP	Father	Frameshift mutation	AD		
56	ANK1	c.2638-2A>C	Splicing	LP	Mother	Splicing mutation	AD		
57	SPTB	c.5179-1G>A	Splicing	P	Spontaneous mutation	Splicing mutation	AD		
58	SPTA1	c.3209G>A	p. R1070Q	LP	Father	Missense mutation	AD/AR	SLC4A1	VUS
		c.5410C>T	p. L1804F	VUS	Mother	Missense mutation	AD/AR		
		c.2807C>T	p. A936V	VUS	Mother	Missense mutation	AD/AR		
59	SPTB	c.507_514del	p. R170Tfs*22	P	Father	Frameshift mutation	AD		
60	ANK1	c.940C>T	p. R314X	P	Mother	Nonsense mutation	AD		
61	ANK1	c.127-3C>G	Splicing	P	Spontaneous mutation	Splicing mutation	AD		
62	SPTB	c.256delC	p. R86Afs*54	P	Spontaneous mutation	Frameshift mutation	AD		
63	SPTB	Deletion of a large fragment of chr 14	deletion	LP	Spontaneous mutation	Deletion mutation	AD		
64	SPTB	c.440T>C	p. L147P	VUS	Mother	Missense mutation	AD		

Sequence variants were interpreted following recommendations from the American College of Medical Genetics and Genomics (8), and identified variants were described using standard terminology: P, Pathogenic; LP, likely pathogenic; VUS, variant of unknown significance; AD, autosomal dominant; AR, autosomal recessive. All variants are heterozygous unless otherwise noted.

### Genotype-phenotype correlation

Almost all patients presented with jaundice, anemia, and splenomegaly, with varying severity. A group analysis of the pathogenicity of different gene variants was performed on 64 patients (Table 3). Spherocytes were observed in the peripheral blood in 21 cases (33%), with >10% spherocytes observed in only one *ANK1* case. Anemia was the primary symptom, including mild anemia (Hb 90–120 g/L) in 8 (13%), moderate (Hb 60–90 g/L) in 38 (59%), severe (Hb 30–60 g/L) in 15 (23%), and very severe (Hb <30 g/L) in 1 cases (2%). However, no statistical differences in hemoglobin levels, MCV, MCH, MCHC, or reticulocytes were observed across variant groups. Bilirubin levels were remarkably elevated in patients with HS variants, and those with *SPTB*-HS had significantly higher bilirubin levels, including total bilirubin (*p* = 0.033) and indirect bilirubin (*p* = 0.018) compared to those with *SPTA1*-HS. A total of 62 HS patients (except for 2 *ANK1*-HS) completed the traditional OF test. The results showed that the positivity rates in those with *SPTB*-HS, *ANK1*-HS, *SLC4A1*-HS, and *SPTA1*-HS were 85% (22/26), 88% (22/25), 60% (3/5), and 33% (2/6), respectively. Moreover, *ANK1* variants displayed reduced resistance to lysis at varying NaCl concentrations in comparison to those with the *SPTA1* variants (*p* = 0.047). Of the eight patients who underwent splenectomy, five (63%) had *ANK1* variants, two (25%) had *SPTB* variants, and one (12%) had an *SLC4A1* variant. None of the patients received splenic embolization or underwent partial splenectomy. The hemoglobin levels of HS patients with *SPTB*, *ANK1* and *SLC4A1* variants improved significantly following splenectomy (*p* < 0.01).

**Table 3:** Laboratory testing results of 64 HS children with different gene variants.

	SPTB (*n* = 26)	ANK1 (*n* = 27)	SLC4A1 (*n* = 5)	SPTA1 (*n* = 6)	*P* value
Spherical erythrocytes (*n*, %)	9 (35%)	8 (30%)	3 (60%)	1 (17%)	
Hb (g/L), [120–158]	77 (52–126)	74 (28.2–114)	70 (44–81)	67.5 (51–83)	0.484
MCV (fl), [77–92]	83.1 (74.2–107.4)	83.2 (75.4–102.8)	81.3 (79.2–84.9)	84.3 (75.4–94.4)	0.853
MCH (pg), [26–34]	27.9 (25.1–36.1)	27.6 (24.9–33.2)	30 (27.6–31.7)	28.2 (23.7–30.4)	0.364
MCHC (g/L), [309–359]	341 (296–367)	331 (280–358)	355 (331–373)	333 (302–369)	0.056
Ret (%), [0.50–2.50]	10.91 (5.49–21.01)	13.2 (1.33–21.53)	10.4 (2.43–12.43)	7.28 (0.96–33)	0.197
T-Bil (umol/L), [3.40–20.50]	66.08 (34.42–639.26)	55.3 (21.36–201.00)	51.79 (31.18–652)	20.35 (7.12–68.84)	0.049*
I-Bil (umol/L), [0.00–17.10]	62.71 (31.01–167.44)	49.2 (17.56–183.37)	43.52 (26.02–101.00)	17.18 (5.96–61.82)	0.027*
OFT (%, hemolysis begin) [0.42–0.46]	0.5 (0.4–0.66)	0.52 (0.42–0.66)	0.48 (0.36–0.52)	0.4 (0.4–0.6)	0.033*
OFT (%, hemolysis complete) [0.32–0.36]	0.34 (0.3–0.4)	0.32 (0.28–0.38)	0.32 (0.28–0.36)	0.32 (0.28–0.38)	0.467
Splenectomy (*n*, %)	2 (8%)	5 (19%)	1 (20%)	0 (0%)	
Hb after Splenectomy (g/L)	116 (107–126)	115 (106–124)	106	—	

Ret, reticulocyte; T-Bil, total bilirubin; I-Bil, indirect bilirubin.

*Kruskal–Wallis test.

## Discussion

Hereditary spherocytosis is based on the pathophysiological effects of defects in genes encoding for one or more of the major RBC cytoskeleton and (trans)membrane proteins: *ANK1*, *SPTB*, *SPTA1*, *SLC4A1,* and *EPB42* (1). HS exhibits great heterogeneity in disease severity among patients, who may be virtually asymptomatic or require frequent transfusions in early childhood. Most patients with HS have mild symptoms, and up to 20%–30% have a purely compensated hemolysis due to a balance between reticulocyte production and red cell destruction (1, 12). Thus, the incidence may be underestimated. In this study, we reported 64 children with HS with an age of disease onset ranging from immediately after birth to 12 years, with >70% of patients having disease onset before 1 year of age. However, the median age at diagnosis was 3.5 years, suggesting that clinical manifestations were not specific and timely to the diagnosis. Conversely, only a third of patients had a family history of HS, also making it difficult to diagnose. Due to the inheritance patterns, some patients had spontaneous mutations, while others showed autosomal recessive inheritance patterns. It is speculated that there may also be incomplete penetrance and variable expressivity among family members (13), so more laboratory tests are needed to diagnose HS.

Currently, the diagnosis of HS mainly relies on the biochemical hemolysis parameters, spherocytes on morphology, and functional testing, such as the OF test, eosin-5-maleimide (EMA) binding test, and membrane protein defects with the SDS-PAGE (14, 15). The drawbacks of the peripheral blood erythrocyte morphology and osmotic fragility test lie in the lack of sensibility and specificity, as other congenital red blood cell defects or hemolytic anemias, may also yield positive results. In our study, spherocytes were observed in the peripheral blood in only a small number of patients, with >10% spherocytes seen in only one *ANK1* case. The determination of the membrane skeleton protein involves the extraction of cell membrane proteins using polyacrylamide gel electrophoresis, which is very intricate and has not been widely promoted in clinical practice. The EMA binding test is widely recognized as the most convenient, sensitive, and specific diagnostic method for diagnosing HS. The combination of this test with other red blood cell osmotic tests has been recommended to enhance diagnostic sensitivity (15). However, a shortcoming of the EMA binding test is the lack of normal controls and a universal reference range for HS (16). Existing research on HS indicates that patients with HS who have an ankyrin protein deficiency have low sensitivity in the EMA binding test (17). Similarly, other red blood cell genetic disorders may also show reduced EMA binding levels. Additionally, flow cytometers are not available in all routine diagnostic laboratories, which also restricts their application.

Recently, genetic testing has shown tremendous potential in the diagnosis of HS. Many researchers have reported that patients with HS could benefit from an early diagnosis via genetic testing. In fact, variations in HS-related genes were identified at a high rate, but molecular defects are significantly heterogeneous (6, 18). Autosomal dominant inheritance (AD) and autosomal recessive inheritance (AR) account for 75% and 25% of HS cases, respectively (19), with *ANK1*, *SPTB*, and *SLC4A1* variants being the most dominant inheritance patterns. Conversely, the *SPTA1* and *EPB42* defects are often recessive or spontaneous mutations, with *ANK1* variants being the most common, followed by *SPTB* (1, 20). Research on the correlation between genotype and phenotype is still insufficient in HS patients, and it is currently controversial. In this study, there were 27 cases (42%) of *ANK1* variants and 26 (41%) of *SPTB* variants, accounting for over 80% of all variants, which is consistent with previous reports. Since *SLC4A1*-HS often onsets during adulthood and presents with mild symptoms, the true prevalence of *SLC4A1*-HS may have been underestimated. Unexpectedly, more than half of the cases were spontaneous mutations and almost all patients exhibited an autosomal dominant inheritance pattern, which may explain the low positive rate of family history. Frameshift, nonsense, and missense mutations are the most common variants. We found 59 variants that have not been reported before. According to the ACMG criteria, 28 novel variants were considered pathogenic, 20 were likely pathogenic, and 11 were unknown. The *ANK1* c.127-3C>G and *SPTB* c.4873C>T were found together in two patients, and the latter has been defined in previous literature as a high-frequency mutation (21).

Herein, we report a significant difference in clinical phenotypes based on the underlying genetic variations. Overall, patients with *SPTB*-HS and *ANK1*-HS had the most severe symptoms, while those with *SPTA1*-HS presented a mild phenotype. Bilirubin levels were remarkably elevated in those with *SPTB*-HS compared to those with *SPTA1*-HS. As mentioned above, cholelithiasis was found in 3 patients with *SPTB* variants, while it was not detected in patients with *SPTA1* variants. In addition, *ANK1*-HS also presented more cholelithiasis and elevated bilirubin levels. This indicates that the level of bilirubin is an important cause of cholelithiasis in these patients, yet it is not the sole determinant. The positive rate of the OFT in *SPTB*-HS and *ANK1*-HS was also higher, and *ANK1*-HS displayed reduced resistance to lysis at varying NaCl concentrations in comparison to *SPTA1*-HS. Previous studies have shown that certain interactions within the ankyrin complex of the cytoskeleton play a crucial role. Specific variants that interfere with these interactions have been found to lead to significant disruptions in cytoskeleton assembly or function, ultimately resulting in a more pronounced phenotype (22). Additionally, the need for blood transfusions was more common in patients with *SPTB* and *ANK1* variants, especially red blood cell transfusion dependence. Patients with *ANK1*-HS and *SPTB*-HS were more likely to undergo splenectomy than other patients. The results of our study are consistent with those of van Vuren, A's research, which reported that variants in *ANK1* and *SPTB* may lead to more severe HS phenotypes compared to variants in *SPTA1* (7).

We analyzed variation type and found *ANK1* and *SPTB* non-missense variations increased and might lead to truncated proteins and loss of expression from the affected allele. This disruption of cytoskeleton function was deemed more deleterious than decrease in the quantity of normally formed protein. Besides, the production of α-spectrin was reported to be three to four folds higher than that of β-spectrin in healthy individuals (23, 24). Considering that *SPTA1*-HS is mostly autosomal recessive owing to dosage compensation effects, most patients with *SPTA1*-HS only exhibit significant clinical symptoms when homozygous or compound heterozygous mutations occur, resulting in milder phenotype compared to other gene variants. Studies showed patients with low expression of the α*^LELY^* allele in *SPTA1* generally exhibit milder clinical symptoms even in a homozygous state, making them difficult to identify (25). Another common *SPTA1* splicing mutation, where the α*^LEPRA^* allele converts to an ineffective variant of *SPTA1*, results in patients who do not require transfusions and benefit more from splenectomy (26). Conversely, patients with combined mutations of the trans-α*^LEPRA^* and α*^PRAGUE^* alleles have been reported to have fatal cases (27). This likely explains the phenotypic variability seen in patients with identical pathogenic HS variants is plausibly attributed to the effects of concomitant variants in modifier genes. However, more research is needed to confirm this theory. *EPB42* defects are often recessive or spontaneous mutations, and Peters et al. reported *EPB42*-knockout mice appeared to have the mildest phenotype characterized by a nearly intact membrane skeleton (28). This may be the reason for the low incidence of *EPB42*-HS.

The distribution of different variant regions in membrane proteins was also considered as a potential factor depending on the severity. Our findings revealed variants distributed across the entire gene. Previous research has indicated that patients with *ANK1*-HS variants in the spectrin-binding domain exhibit the most severe anemia among affected individuals (22). Moreover, loss-of-function variants in the ZU5 subdomain have also been documented (29, 30). Park et al. suggested that patients with variants in the c.2482-4149 region of the spectrin-binding domain of *ANK1* may manifest a more severe phenotype (22). Interestingly, individuals in our cohort did not exhibit a higher severity of anemia despite variant presence in this region. Because no statistical differences across variant groups were observed in hemoglobin levels, MCV, MCH, MCHC, or reticulocytes in our cohort, we conclude that categorization in different genetic subgroups is insufficient to precisely predict HS phenotype. There are several limitations to this study. Firstly, it is a retrospective report. Some patients are transfusion-dependent, which may obscure the laboratory testing results and likely underestimate the severity of the disease. Secondly, it has a small sample size. By increasing the sample size and conducting further research, a more intricate and thorough outcome will be obtained. Thirdly, the incidence of childhood splenectomy may be underestimated in this patient population due to the young age of many participants who may not have had the time to develop complications that would necessitate a splenectomy. Moreover, splenic embolization and partial splenectomy are not available in our center. Stratifying patient age in future studies may help reduce data bias to some extent. In our study, there was a broad phenotypic variability among patients in each genetic subgroup. To further identify the genotype-phenotype correlations in HS, a functional assay of concomitant variants in modifier genes, variant regions, and the pathogenicity of VUS, family members variants, and pedigree analysis are required. New parameters such as mean sphered corpuscular volume (MSCV), Ret/immature reticulocyte fraction (IRF), and mean reticulocyte volume (MRV), were demonstrated to be sensitive parameters for HS detection (31). New insights may be discovered by combining these into diagnostic algorithms and phenotype studies. Although HS is a hereditary disorder cannot be prevented, the progression of molecular diagnostics enables the discovery of new variants and allow for a comprehensive genetic analysis and definitive diagnosis in patients suspected of HS without typical clinical presentation, family history, and hematologic laboratory tests. Through extensive exploration into the relationship between phenotype and genotype in HS, early genetic testing can be utilized in the assessment of disease risk and the selection of appropriate treatments. It can also aid in distinguishing spontaneous mutations and autosomal recessive inheritance cases, thus facilitating genetic counseling for affected families, which may also be beneficial for investigating the specific underlying pathophysiological mechanisms of individuals or families with HS.

## Conclusion

In summary, we reported our findings in 64 children with HS with different red cell membrane cytoskeleton protein gene variants and analyzed the clinical phenotypes stratified by variant types, with *ANK1* and *SPTB* variants being associated with the most severe disease and *SPTA1* variants with the mildest. To explore the correlation between clinical and mutational features of HS patients, genetic testing is proposed in patients without a family history or who are difficult to diagnose with routine laboratory tests, which may also provide references for clinical treatment and genetic counseling.

## Data Availability

The datasets generated during and/or analysed during the current study are not publicly available. However, datasets are available from the corresponding author on reasonable request.
